# A high-throughput inhibitor screen for the erythrocyte plasma membrane Ca^++^ ATPase

**DOI:** 10.1371/journal.pone.0352460

**Published:** 2026-06-25

**Authors:** Jonathan Chu, Shreyas Annaswamy, Sanjay A. Desai

**Affiliations:** The Laboratory of Malaria and Vector Research, National Institute of Allergy and Infectious Diseases, National Institutes of Health, Rockville, Maryland, United States of America; UC Riverside: University of California Riverside, UNITED STATES OF AMERICA

## Abstract

The ubiquitous and conserved plasma membrane Ca^++^ ATPase (PMCA) actively extrudes Ca^++^ from eukaryotic cells and maintains a large Ca^++^ transmembrane gradient that permits diverse roles in signaling and cell cycle regulation. Molecular and biochemical studies have linked PMCA to multiple diseases such as sickle cell disease and malaria severity for the PMCA4b isoform on human erythrocytes. Despite its central role in Ca^++^ biology, there are no PMCA inhibitors with sufficient specificity to serve as starting points for therapy development. Because such inhibitors of other ion pumps are critical research tools and are important therapeutics for various diseases, we designed and executed a cell-based high-throughput screen for PMCA inhibitors using human erythrocytes. We miniaturized and optimized a Ca^++^ efflux assay using extracellular Fluo-8, a soluble Ca^++^ sensitive indicator dye. Ca^++^ loading, dye concentration and affinity, hematocrit, and assay temperature were all optimized, providing a robust miniaturized assay for PMCA-mediated Ca^++^ efflux. A screen of >52,000 diverse compounds was executed with readings at 2 time points to permit detection of inhibitors with varying affinity; addition of excess extracellular Ca^++^ helped exclude false positive fluorescence quenchers. A surprisingly low hit rate, ~ 0.01%, suggests that PMCA has relatively few chemical binding sites that interfere with pump activity. As the screen did not yield reproducible hits, a larger screen with more complex chemical libraries is warranted. The optimized assay should enable identification of specific PMCA inhibitors as research tools and potential future therapeutics.

## Introduction

Ca^++^ serves many essential roles in biology as it mediates diverse cellular processes including excitation-contraction coupling, intracellular signaling as a second messenger, and triggering DNA replication and other key steps in cell cycle progression [1–3]. These roles depend on a large transmembrane Ca^++^ gradient at the cell’s plasma membrane, a tightly controlled cytosolic free submicromolar Ca^++^ concentration, and numerous Ca^++^-dependent enzymes and modulators including calmodulin and C2 domain-containing effectors [4–6].

The plasma membrane Ca^++^ ATPase (PMCA) functions as a Ca^++^ extrusion pump and is primarily responsible for maintaining the plasma membrane gradient in single-cell protists, plants, invertebrates and vertebrates including humans [7,8]. This P-type ATPase uses the energy from ATP hydrolysis to drive Ca^++^ extrusion, often maintaining a > 10,000-fold gradient at the plasma membrane. Divergent regulatory requirements for cellular Ca^++^ in various tissues has driven expansion of *pmca* gene copy number, alternative splicing, and differential expression through development [9]. The resulting PMCA isoforms differ in their resting activities, calmodulin affinity and degree of stimulation [10]. PMCA mutations have also been linked to various diseases in humans and animals [11–13]).

Remarkably, despite discovery in the 1960s and purification shortly thereafter [14,15], there are no highly specific chemical inhibitors of PMCA. A few nonspecific small molecule inhibitors have been found and used. Aurintricarboxylic acid was identified in a small screen as having specificity for PMCA4 over PMCA1 and unrelated ATPases [16], but it inhibits other ion channels and enzymes, often with higher affinity than that for PMCA4 [17–20]. Resveratrol was identified as a broad-spectrum PMCA inhibitor after observed effects on Ca^++^ signaling [21], but these effects may be manifest through indirect activation of sarcoplasmic/endoplasmic reticulum Ca^++^ ATPase pumps (SERCAs) or through direct action on other enzymes [22–24]. Although both chemicals are considered pan-assay interference compounds [25], they have provided useful insights in some cases [26,27]. Somewhat more specific peptide inhibitors, termed caloxins [28], have been found, but these agents are low-affinity and susceptible to proteases. This deficiency is further highlighted because other ion pumps have potent and specific inhibitors such as cardiac glycosides for the Na^+^/K^+^ ATPase and omeprazole for the gastric H^+^/K^+^ pump. Importantly, these ion pump inhibitors are important therapeutics for heart failure and arrhythmias [29], cancer [30], acid reflux and H. pylori infections [31]), suggesting that PMCA inhibitors may also prove to be useful chemotherapeutics.

As a step in addressing this deficiency, we now report a novel cell-based high-throughput chemical screen for PMCA inhibitors using human erythrocytes. Erythrocytes are not only a model system where PMCA activity was first identified and cloned, but the specific isoform in these cells (PMCA4b) has also been linked to sickle cell disease and malaria severity [32–34]. Although the few hits found in our screen proved to be false-positives, these studies implicate a low hit rate for PMCA small molecule inhibitors and should stimulate systematic, larger-scale surveys for much needed inhibitors of this ubiquitous Ca^++^ pump.

## Materials and methods

### Materials

O^+^ blood from anonymized human donors was obtained from commercial sources (Interstate Blood Bank, Memphis TN or University of Virginia Blood Bank) after screening for transmissible infectious diseases and leukofiltration to remove white blood cells. Blood was washed in RPMI 1640 supplemented with 25 mM HEPES, 50 µg/mL hypoxanthine, and 1 µg/mL gentamicin, stored at 50% hematocrit (hct) and used within 7 days. Chemicals were obtained from Sigma Aldrich (St. Louis MO) or VWR (Radnor, PA) unless otherwise indicated. The small molecule screening library and resupply of screening hits were from ChemBridge (San Diego, CA).

### Fluorescence kinetics using Fluo-8 AM loaded erythrocytes

Human erythrocytes were loaded with Fluo-8 AM as described previously [35]. Washed cells were resuspended at 3% hematocrit in 150 mM NaCl, 20 mM HEPES, 1.0 mM EGTA, 5 μM Fluo-8 AM, pH 7.4 with NaOH. Fluo-8 AM (AAT Bioquest, Pleasanton, CA) was freshly dissolved from a DMSO stock with vortexing immediately before use. Cells were incubated at 37 °C for 1 h to permit dye uptake and acetoxymethyl (AM) ester hydrolysis by intracellular esterases; hydrolysis is expected to trap the dye in the cell and activate Ca^++^-dependent fluorescence. After subsequent washing at 4 °C, erythrocytes were resuspended in a black clear-bottom 384-well microplate (Greiner Bio-One, Monroe, NC) at 1.5% hct in 150 mM NaCl, 20 mM HEPES, 1.5 mM CaCl2, 0.5 mM EGTA, pH 7.4, with or without 20 μM diflunisal to initiate transport at room temperature with a buffered free extracellular [Ca^++^] of 1.0 mM. The microplate was centrifuged (~ 1000x *g*, 1 min) and fluorescence kinetic recordings were initiated immediately (excitation/emission at 495/10 and 516/10 nm, respectively; BioTek Synergy Neo2 microplate reader, Agilent, Santa Clara, CA).

### Ca^++^ efflux assay

In contrast to typical studies using indicator-loaded cells, the Ca^++^ efflux assay used in these studies utilizes cells loaded with Ca^++^ and tracks efflux of this cation into extracellular buffer containing Fluo-8 [35]. Here, erythrocytes were washed in Buffer A (100 mM KCl, 30 mM MOPS, pH 7.2 with KOH) at 4 °C. The cells were then resuspended at 5% hct in Buffer A supplemented with 500 µM CaCl_2_, 150 µM MgCl_2_, and 500 nM A23187, added prior to use from a 200 µM DMSO stock. The suspension was incubated for 20 min at 37 °C to allow for ionophore-mediated Ca^++^ loading. The ionophore was then removed to restore endogenous membrane Ca^++^ permeability by three washes in Buffer A with 10 g/L lipid-rich AlbumiNZ bovine albumin (MP Biomedicals, Irvine CA) at 4 °C followed by one wash in Buffer A and storage on ice at 10% hct in Buffer A until used. To initiate efflux, a 50 uL suspension of these Ca^++^-loaded cells was added to an equal volume of 2x Dye Buffer (Buffer A + 20 µM EGTA, 0.8 µM Fluo-8 sodium salt (AAT Bioquest), pH 7.2 with KOH) supplemented without or with Na_3_VO_4_, JCE hit compound, or 1% DMSO control at room temperature in a black clear-bottom 384-well microplate (Greiner Bio-One). After mixing with a pipette tip, the microplate was immediately centrifuged to pellet the cells (233 x *g,* 1 min, 4 °C). Fluorescence was recorded at 10 min intervals for up to 12 h (excitation/emission at 495/10 and 516/10 nm; BioTek Synergy Neo2 microplate reader).

### High-throughput screen

The high-throughput screen followed the procedures of the Ca^++^ efflux assay with modifications to permit robust screening results over many 384-well microplates. Most screening days utilized 30 microplates and entailed fresh preparation of 6 L of Buffer A, 570 mL of the A23187-containing Ca^++^-loading buffer, and 3 L of the lipid-rich bovine albumin-containing buffer. The typical 30 microplate single-day screen corresponds to 9600 compounds and 720 wells each for negative and positive in-plate controls. For this scale, 40 mL of packed human erythrocytes were loaded with Ca^++^ and washed to remove A23187 as in the standard Ca^++^ efflux assay before resuspension in Buffer A at 2.67% hct and storage on ice until initiation of efflux in the high-throughput screen. Microplates were spray-cleaned and static-dissipated (Top Gun III, Simco-ION, Aalsvoort, Netherlands) prior to dispense of 20 uL of 4x Screening Dye Buffer (Buffer A + 40 µM EGTA, 0.8 µM Fluo-8 sodium salt (AAT Bioquest), without or with 8 mM Na_3_VO_4_, pH 7.2 with KOH) using a Multidrop Combi+ microplate dispenser (ThermoFisher, Waltham, MA) at room temperature. Microplate columns 1−2 and 23−24 were reserved for negative no addition and positive Na_3_VO_4_ control wells, respectively. Compounds from chemical screening libraries were dispensed by 100 nL pin transfer of a 10 mM DMSO stock using a VP 903 pin tool robot (V&P Scientific, San Diego, CA). Our screening libraries (ChemBridge, San Diego, CA and an in-house collection) were designed and selected based on diversity and drug-like properties with MW ≤ 450 dal and cLogP values ≤ 4.5. Microplates were sealed, vortexed and centrifuged before cooling to 4 °C. To initiate efflux in the screen, 60 µL of the Ca^++^-loaded cell suspension was added to yield a 2% hct suspension in Buffer A + 10 µM EGTA, 0.2 µM Fluo-8 with or without 2 mM Na_3_VO_4_, pH 7.2. Following dispense, each plate was sealed, vortexed and incubated under a heavy metal plate to facilitate rapid, uniform warming to room temperature. After 20 min of acclimation, the plate was centrifuged (233 x *g,* 1 min, 4 °C) and was kept sealed for a room temperature incubation until and between individual timepoint readings at 1 h and 3 h as above. After the second reading, 5 µL of Buffer A + 200 µM CaCl_2_ + 10 µM EGTA was added to yield a free extracellular [Ca^++^] of ~ 11 µM. Plates were again sealed, vortexed and incubated for 2 h before a third reading to detect fluorescence-quenching compounds, which are predicted to have a blunted increase in Ca^++^-dependent Fluo-8 fluorescence.

In-plate controls were used to calculate the Z’ statistic and evaluate assay suitability for high-throughput screening according to:


$$\begin{array}{c} Z^{\prime }=\text{1}-\text{3}(\sigma_{pos}+\sigma_{neg})/({\overset{―}{F}}_{neg}-{\overset{―}{F}}_{pos}) \end{array}$$ (1)

where $\sigma_{pos}$ and $\sigma_{neg}\;$represent the standard deviations of the positive and negative control wells in a given plate; ${\overset{―}{F}}_{pos}$ and ${\overset{―}{F}}_{neg}$ represent the mean fluorescence values for the corresponding plate’s positive and negative control wells, respectively. A value ≥ 0.5 indicates a robust assay suitable for single measurement high-throughput screening and a low false positive rate [36]. A Z’ statistic was similarly calculated for each screening day by tabulating the control well readings from all plates screened on a given day.

Individual well readings at each timepoint were then normalized according to Eq. 2:


$$\begin{array}{c} norm.\;F=\text{1}\text{0}\text{0}*(F_{well}-{\overset{―}{F}}_{pos})/({\overset{―}{F}}_{neg}-{\overset{―}{F}}_{pos}) \end{array}$$ (2)

where *F*_*well*_ represents the individual well’s fluorescence and ${\overset{―}{F}}_{pos}$, *and* ${\overset{―}{F}}_{neg}$ are the mean values for the corresponding plate’s control wells. This normalization assigns values of 0 and 100 to the mean fluorescence values for the plate’s positive and negative controls and yields a normalized % PMCA4b activity for each screening compound. This normalization permitted tabulation of all-points histograms for each timepoint. Histograms were generated using selected linear scale bin sizes and SigmaPlot 14.5 (Grafiti, Hyderabad, India).

### Cell-free assays for fluorescence quenchers

Screening hits were evaluated for false-positive fluorescence quenching activity with a cell-free assay that closely follows the Ca^++^ efflux assay without addition of erythrocytes. Here, JCE compounds were added at indicated concentrations to Buffer A + 20 µM EGTA + 0.8 µM Fluo-8 sodium salt and vortexed to mix; matched negative controls were performed and used either no addition or 1% DMSO. To quantify quenching, 50 µL of these solutions were added to 50 µL of Buffer A with 0, 10, or 20 µM CaCl_2_ in individual wells of a 384-well microplate. The plate was centrifuged and read as above. Readings were normalized to 100% for matched DMSO controls at each [Ca^++^]. Greater quenching with added Ca^++^ suggests a compound that interferes with productive Ca^++^ and Fluo-8 interaction.

## Results

### Assay miniaturization for high-throughput screening

**Fig 1:**
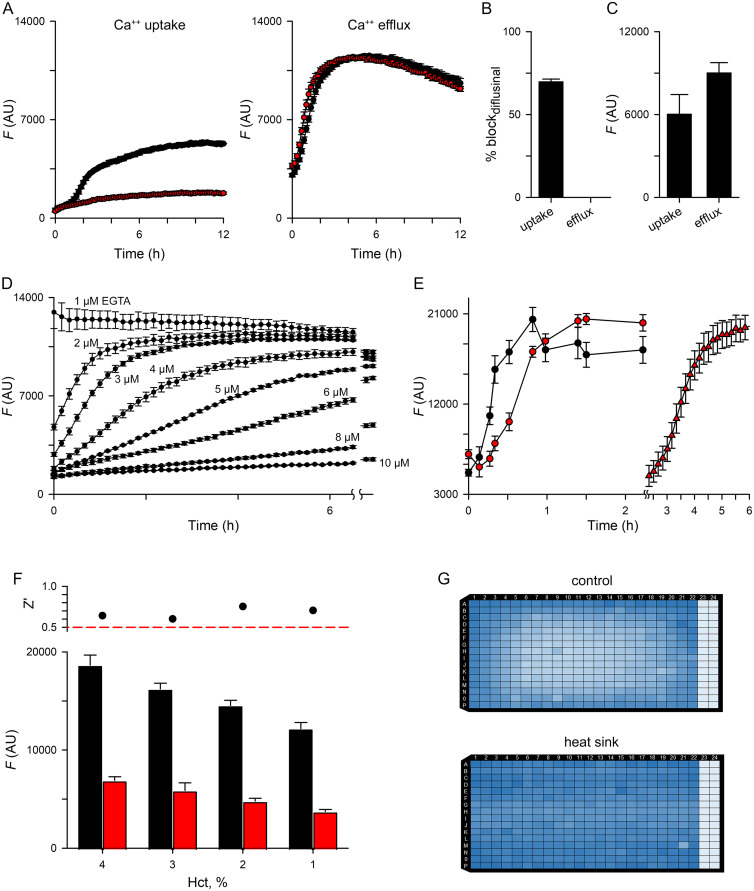
Optimization of Ca^++^ efflux assay for high-throughput screening. **A)** Kinetics of Ca^^++^^ uptake using Fluo-8 AM-loaded cells compared to Ca^++^ efflux into buffer with extracellular Fluo-8 (left and right panels, respectively). Black and red symbols represent addition of 0 and 20 µM diflunisal, a specific OAT inhibitor. The uptake assay is compromised by dye efflux through OATs while the efflux assay is unaffected. **B)** Mean ± S.E.M. %block by 20 µM diflunisal from kinetic uptake and efflux experiments as in panel **A.** %block was calculated as the reduction in fluorescence increase at 6h relative to control. **C)** Mean ± S.E.M. signal in both assays, determined as the difference between the maximum and initial fluorescence values. **D)** Ca^++^ efflux kinetics with indicated concentrations of EGTA added to the efflux buffer containing 0.2 µM Fluo‑8. Note that low [EGTA] improves efflux signals by reducing initial fluorescence associated with trace Ca^++^ in the buffer; high [EGTA] outcompetes Fluo-8 for Ca^++^ binding and compromises signal development. **E)** Efflux kinetics at 37 °C and room temperature (black and red symbols). Ca^++^-loaded cells were either used for efflux kinetics immediately (circles) or stored on ice for 2.5 h before use (triangles). Note the modestly slower kinetics at room temperature than at 37 °C and a preserved efflux signal without an increase in initial fluorescence upon ice-storage of loaded erythrocytes. **F)** Mean ± S.D. fluorescence without or with 2 mM Na_3_VO_4_ addition (black and red bars, respectively) to wells with indicated hematocrits (hct) in room temperature efflux experiments (*n* = 36 wells each, read at 2 **h)**. Top graphic shows Z’ statistic calculated using mean and S.D. values from the main graphic. Red dashed line, Z’ = 0.5, represents the minimum value recommended for single-point inhibitor screens [36]. **G)** Heat maps showing 384-well microplate readings in inhibitor screen plates without and with incubation of screening plate under a heavy metal sheet (heat sink). Columns 23 and 24 contain in-plate 2 mM Na_3_VO_4_ control and have reduced fluorescence. Note, the lighter central wells in the control, indicative of slowed warming and reduced spuriously reduced Ca^++^ efflux; this artifact is largely abolished by the heat sink. A screening hit is apparent in the bottom plate (well M21).

Transmembrane Ca^++^ transport studies typically use cells loaded with fluorescent Ca^++^-sensitive indicator dyes conjugated to hydrolyzable acetoxymethyl (AM) esters to track Ca^++^ uptake [37–39]. While conjugation with nonpolar AM esters permits dye loading and intracellular trapping through AM ester hydrolysis by intracellular esterases, several problems with this approach have been reported. These include variable dye loading efficiency, biased loading into organelles, toxicity from AM ester breakdown products, and dye efflux from cells [40–42]. Consistent with this, we reported that Ca^++^ indicator dyes undergo rapid efflux from human erythrocytes via organic anion transporters (OATs) [35]. Here, we confirmed that the Ca^++^-sensitive Fluo-8 dye also exhibits unwanted efflux via OATs as diflunisal, a specific OAT inhibitor, aborted fluorescence increases in experiments with loaded human erythrocytes (Fig 1A, left panel). Rather than reflecting Ca^++^ uptake as intended, this fluorescence kinetic, therefore, reflects Fluo-8 efflux from erythrocytes and binding to extracellular Ca^++.^

To address the limitation, we reversed the transport experimental design to enable measurement of Ca^++^ efflux. To achieve this, we loaded erythrocytes with Ca^++^ and measured the efflux kinetic with soluble Fluo‑8 indicator added to the extracellular buffer. Because this design uses the Fluo-8 salt instead of the AM ester-conjugated dye, it circumvents problems associated with dye loading and AM ester cytotoxicity. Importantly, we found that the resulting Ca^++^ efflux signal is not affected by diflunisal (Fig 1A, right panel; *P <* 10^−4^ for comparison of diflunisal block in uptake and efflux assays, Fig 1B). As the extracellular Fluo-8 concentration can be precisely controlled, we also found that the efflux assay can be tuned for a larger signal (Fig 1C), an advantageous feature for high-throughput screening.

To improve the fluorescence signal-to-background ratio, we next added the non-fluorescent Ca^++^ chelator EGTA together with Fluo‑8 to tune the apparent Ca^++^ affinity. With addition of only 1 µM EGTA, Fluo‑8 was bound by contaminating Ca^++^ in the extracellular buffer and an efflux signal could not be recorded (Fig 1D). Increasing concentrations of EGTA abolished initial fluorescence to yield a maximal signal-to-background ratio at 3 µM EGTA. Further increases in [EGTA] reduced the signal as it lowers the apparent Ca^++^ affinity; 10 µM EGTA eliminated efflux-associated fluorescence as it outcompeted Fluo-8 for binding to effluxed Ca^++^ (Fig 1D).

Although PMCA is generally assumed to have a physiologically relevant temperature optimum around 37 °C, we recognized that elevated temperatures could adversely affect our high-throughput screen through various factors such as evaporation in small-volume microplate wells. We therefore tracked PMCA4b-mediated Ca^++^ efflux at room temperature and found a comparable efflux-associated signal with modestly slower kinetics (Fig 1E, red circles) when compared to 37 °C (black circles). Importantly, efflux was abolished at 4 °C but the pump remained functional for at least 2.5 h as efflux could be activated by raising the temperature (red triangles); the fluorescence plateau achieved by these stored cells was indistinguishable from that observed with cells that were used immediately after Ca^++^ loading.

To reduce usage of human blood and streamline the screening process, we next evaluated the fluorescence signal at reduced hematocrits and calculated the assay’s Z’ statistic, a parameter that relies on measurements with positive and negative controls to evaluate assay suitability for high-throughput screening [36]. We used measurements without and with 2 mM VO_4_^−3^, a nonspecific ATPase inhibitor that abolishes PMCA4b-mediated efflux, as controls to calculate Z’ values (Fig 1F, bottom, black and red bars respectively). Here, we examined a range of hematocrits because commercial human blood products represent a significant expense for these screens. Decreasing the hematocrit from 4% to 1% reduced the absolute fluorescence signal but did not compromise the Z’ statistic (Fig 1F, top and Eq 1); the Z’ remained consistently above 0.5 (red dashed line), a value commonly used to justify single measurement high-throughput screens.

### Execution of high-throughput chemical screen for PMCA inhibitors

With these optimized conditions, we initiated high-throughput screens for specific PMCA4b inhibitors, which may be research tools and lead compounds for therapy development. Our procedure entailed dispensing ice-cold Ca^++^-loaded cells into 384-well plates at 4 °C and subsequent warming to room temperature to initiate efflux and permit timed, sequential reading of microplates. Our early experiments revealed edge effects on efflux kinetics because the middle of each microplate warmed more slowly than edge wells (Fig 1G, top). We therefore instituted plate incubation under a heavy metal sheet used as a heat sink to ensure rapid and uniform plate warming to room temperature and initiation of efflux (Fig 1G, bottom).

**Fig 2:**
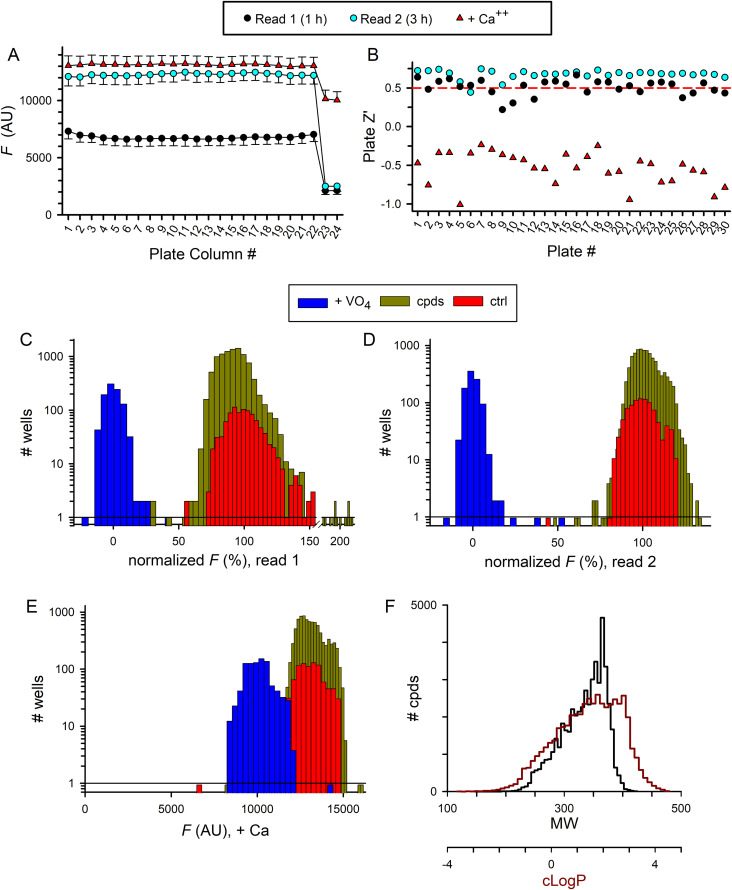
A cell-based fluorescent screen for novel PMCA4b inhibitors. **A)** Mean ± **S.**D. fluorescence for each column of microplates from a single day of PMCA inhibitor screens. Note that values in columns 1 through 22 are indistinguishable at each Read, excluding noticeable plate position effects. Columns 23 and 24 contained in-plate vanadate positive controls and yielded nearly identical readings. Fluorescence increases from Read 1 to Read 2, indicating ongoing PMCA-mediated Ca^++^ efflux between these time points. After exogenous Ca^++^ addition, fluorescence increases to a much greater extent in columns 23-24. **B)** Z’ statistics calculated for each plate from this screening day at each Read timepoint using the in-plate controls. Red dashed line indicates the threshold Z’ of 0.5 for single reading high-throughput screens. C-E) Log scale histograms of all microplate well readings on this day for Reads 1, 2, and 3 respectively. Values are normalized to 0 and 100 for the mean of each plate’s negative and positive controls. **F)** Histogram of molecular weights (MW, black trace) and cLogP (red trace) for a 50,000 compound library used in these screens; a smaller collection of natural product-like compounds screened against PMCA is not included in this histogram as these molecular properties are unavailable.

We then completed a screen of a > 52,000 small molecule library using this assay. Fig 2 shows the analysis of results from a single day where 9600 chemicals were tested in 30 microplates containing individual compounds in each well of plate columns 3–22. Columns 1 and 2 were empty and reserved for inhibitor-free negative controls while columns 23 and 24 were dispensed with a final 2.0 mM Na_3_VO_4_ to yield a fully-blocked PMCA4b positive control. Each plate was read at 1 h and 3 h after initiation of room-temperature efflux. The early 1 h reading was designed to detect relatively weak inhibitors that exhibit slower efflux kinetics that still reach the same fluorescence plateau as uninhibited controls before the 3 h reading. The more stringent 3 h reading was designed to identify only the most potent inhibitors.

We also instituted addition of Ca^++^ after the second reading to distinguish true PMCA4b inhibitors from false-positive fluorescence quenchers: Ca^++^ addition is expected to increase the signal with PMCA4b inhibitors as it overcomes blocked Ca^++^ efflux but the increase would be blunted in wells containing fluorescence quenchers.

Fig 2A shows the mean and standard deviations of each column from all 30 microplates screens on the selected screening day and at each of the three readings. Upon averaging, the negative controls and compound wells were indistinguishable, consistent with a robust screen having a low false positive rate and acceptably low hit rate. The Na_3_VO_4_ positive controls exhibited lower fluorescence values in the first two readings and a much greater increase after Ca^++^ addition, as we predicted for wells where PMCA-mediated efflux is blocked.

For each screening plate, we used measured fluorescence from the 32 negative and 32 positive control wells to determine each plate’s Z’ statistic for each read (Fig 2B). Consistent with a relatively early measurement in the efflux kinetic, the Z’ values for Read 1 were lower than those for Read 2 where efflux was allowed to reach saturation fluorescence in the negative controls. Both readings yielded Z’ statistics near or exceeding the Z’ = 0.5 threshold, which further established assay robustness for high-throughput screens. Because the difference between the controls is largely abolished by Ca^++^ addition, the Z’ statistic for Read 3 is much lower, ranging from −1.0 to −0.23.

We next tabulated the results from all wells into log-scale histograms after normalization of fluorescence values to the in-plate controls using Eq. 2. Fig 2C, D, and E show the all-points histograms for Reads 1–3, respectively. As expected, the positive and negative controls exhibit distinct bell-shaped profiles centered at 0 and 100%. At each read timepoint, the compound histogram closely resembles the negative control, but a few wells fall outside the range of negative control readings. Compounds with low normalized fluorescence values were considered hits that may block PMCA4b. Fig 2F shows the all-points histogram of molecular weights and calculated LogP (cLogP) values for the primary library used in our screens; these compounds have drug-like properties.

### Secondary studies with screening hits

**Fig 3:**
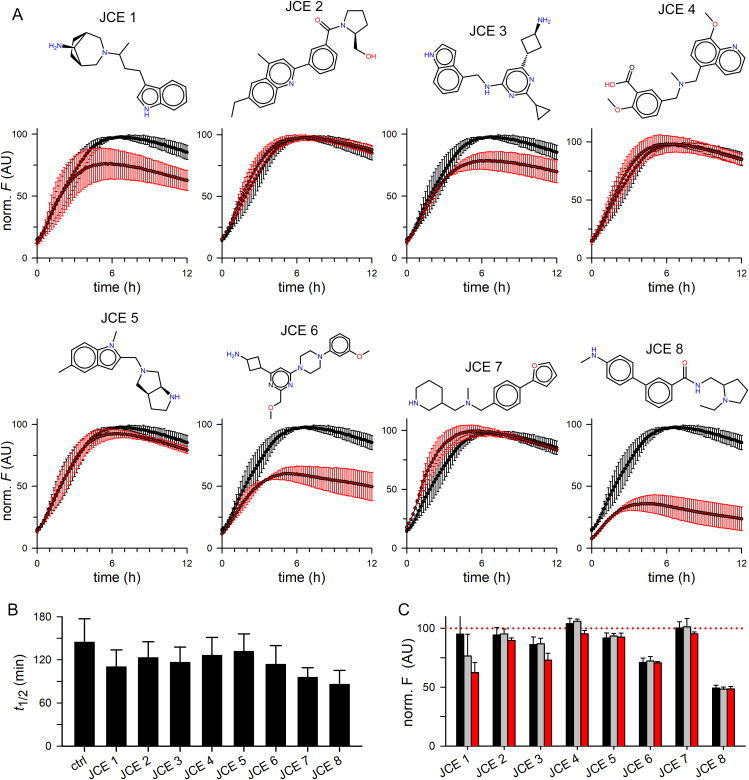
Secondary studies with hits from the PMCA inhibitor screen. **A)** Structures and Ca^++^ efflux kinetics with 8 screening hits, obtained in resupply. Kinetics are shown with 12.5 µM of each hit (red symbols) after normalization of matched inhibitor-free control (black symbols) to 100 for the fluorescence maximum. Symbols represent mean ± **S.**E.M. of 3 independent trials each. **B)** Mean ± **S.**E.M efflux halftimes, *t*_*1/2*_, without (ctrl) or with 12.5 µM screening hit, calculated from efflux kinetics as in panel **A.** None of the hits increased *t*_*1/2*_ as expected for reversible inhibitors. **C)** Fluo-8 fluorescence in the presence of 12.5 µM screening hit and 0, 5 µM or 10 µM added Ca^++^ (black, grey, and red bars, respectively). Values are normalized to 100 for matched controls without inhibitors; lower values implicate nonspecific fluorescence quenchers. Mean ± **S.**E.M of 3 independent trials.

We selected 8 compounds for secondary studies based on low normalized fluorescence values in Reads 1 and 2 followed by a significant increase in the post-Ca^++^ addition Read 3. Several other hits that met our criteria were, unfortunately, no longer available in resupply. Fig 3A shows Ca^++^ efflux kinetics for each of the 8 hits, which we named JCE 1 through JCE 8. Three independent trials with each compound at 12.5 µM, the estimated concentration in our high-throughput screen, were averaged after normalization to a matched DMSO control’s maximum fluorescence. JCE 8 was the most potent hit; several other hits—JCE 1, JCE 3 and JCE 6—also reduced fluorescence development with lower plateaus. It is unclear why JCE 2, JCE 4, JC5, and JCE 7 failed to reproduce as these secondary studies followed closely the experimental design of the high-throughput screen. Although our screen’s high Z’ values predict a low false-positive rate (Fig 2B), the unexpectedly low hit rate may bias toward identification of false-positives by yielding a low positive predictive value [43]. It is possible that these false-positives represent compound degradation in our screening library collection or erroneous resupply from vendors.

Reversible inhibition of PMCA-mediated Ca^++^ efflux by small molecules is expected to slow the efflux kinetic without reducing the fluorescence plateau when compared to inhibitor-free controls because reversible inhibitors permit nonzero Ca^++^ efflux that continues until the same final extracellular [Ca^++^] is reached. To explore whether any of our hits exhibit this behavior, we estimated the time to half-maximal fluorescence, *t*_*1/2*_, for each compound (Fig 3B, *n* = 3). No hit increased this halftime relative to matched DMSO controls.

We then evaluated each hit in cell-free fluorescence measurements using Fluo-8, the Ca^++^-sensitive dye used in our screen, without and with addition of 5 µM or 10 µM Ca^++^, values selected based on efflux from Ca^++^-loaded cells in our screen (Fig 3C, *n* = 3). Normalization to DMSO controls revealed reduced fluorescence associated with each JCE compound active in our cell-based assays, implicating fluorescence quenching as the primary mechanism of reduced signals in our screen. JCE 8 produced > 50% quenching independent of [Ca^++^]; JCE 6 also exhibited Ca^++^-independent quenching. In contrast, JCE 1 and JCE 3 produced greater fluorescence quenching with higher [Ca^++^], suggesting that these compounds interfere with productive interactions between Fluo-8 and Ca^++^, either directly or indirectly. Unfortunately, these findings suggest that none of our selected hits target PMCA-mediated Ca^++^ efflux from human erythrocytes.

## Discussion

We report the miniaturization and optimization of a Ca^++^ efflux assay for the plasma membrane Ca^++^ ATPase (PMCA), the primary mechanism used to establish a ≥ 10,000-fold Ca^++^ gradient at eukaryotic plasma membranes. Our assay avoids use of costly and hazardous radioactive ^45^Ca^++^; it also circumvents AM ester conjugated Ca^++^-indicator dyes, which are toxic to some cellular activities and prone to various artifacts [40,41]. By loading Ca^++^ into cells and tracking efflux with extracellular Fluo-8 in EGTA-buffered solutions, we achieved a robust efflux-specific signal not subject to OAT-mediated dye transport [42]. We also established that the PMCA in Ca^++^-loaded cells remains dormant at 4 °C but can be restored to an active state at room temperature. Our studies also provide conditions for an optimized signal-to-noise ratio. These features should enable future high-throughput screens for PMCA inhibitors and agonists.

We then executed a 52,000 small molecule screen for PMCA inhibitors as useful research tools and potential therapeutics for malaria, sickle cell, and other diseases [32,33]. Although our screen did not find reproducible inhibitors, our methodology should prove broadly useful for larger screens. As these studies establish a low hit rate using a diverse small molecule library, we propose that the PMCA pump may have relatively few chemical binding sites that lead to inhibition when occupied. This may account for the absence of specific inhibitors for this ATPase, which is indeed surprising given that it has been studied for decades and other ATPase ion pumps have potent and specific inhibitors used in both research laboratories and medical clinics [29–31]. PMCA inhibition by aurintricarboxylic acid and resveratrol indicate that effective block by small molecules is indeed possible [16,21]. We note that chemicals in our screening library were relatively small with molecular weights ≤ 450 dal and cLogP values ≤ 4.5 (Fig 2F). While these are desirable drug-like properties [44], we note that digitalis, a specific Na^+^/K^+^ ATPase pump inhibitor, has a molecular weight of 781 dal and is based on an estrogen-like steroid scaffold [45]. Our findings suggest that chemicals with higher molecular weights and/or lipophilic scaffolds may produce more specific inhibitory interactions with P-type ATPases; a lipophilic scaffold may also facilitate access to inhibitor binding sites along the pump-lipid bilayer interface.

A larger chemical screen, possibly using more diverse chemical libraries such as natural product libraries, is warranted as specific PMCA inhibitors are expected to be critical research tools and potential therapeutics. When found, we envision that derivatives could be made that have specificity for each of the 4 *pmca* gene products in humans. Such inhibitor compounds would be useful for understanding PMCA roles and regulation in diverse tissues and the biological purposes of multiple PMCA splice variants. For example, noncoding mutations in the human *pmca4b* locus are a strong predictor of malaria disease severity [34]. These mutations appear to reduce erythrocyte PMCA expression and have been suggested to compromise intracellular parasite development by altering infected cell Ca^++^ homeostasis [46,47]; whether altered malaria severity results directly from changes in intracellular [Ca^++^] or indirectly through effects on cell volume, deformability, or other parameters is unclear [48]. Our improved assay directly reports on Ca^++^ transport, is not subject to artifacts associated with fluorescent dye flux, and should facilitate discovery of highly specific PMCA4 inhibitors. This assay and such inhibitors will help address these and other questions relating to PMCA role in cellular and disease processes.
