# Supplementary material for: A high-throughput inhibitor screen for the erythrocyte plasma membrane Ca++ ATPase
Source: PLoS One. 2026 Jun 25;21(6):e0352460. doi: 10.1371/journal.pone.0352460 (PMC13298757; doi:10.1371/journal.pone.0352460)
Supplement: Attachment — Submitted filename: Response to Reviewers 060926.docx [file pone.0352460.s003.docx]

*Editor:The paper is ready for publication, however, as pointed out by Reviewer 2, the title is misleading in that the study does not involve Sickle Cell or malaria research (although the applications are clear). Please consider a more appropriate title that relates to the actual findings in this study.*

*Reviewer #1: This is a clear-cut, easy to read, interesting and potentially useful original manuscript on the development and optimisation of a novel cell-based high-throughput chemical screen for PMCA enzyme inhibitors using human erythrocytes as model system, based on a Ca2+ efflux assay for the plasma membrane Ca2+ ATPase (PMCA). The work has been carefully made and edited, and this manuscript doesn’t have any evident suggestion for improvement.*

*Reviewer #2: I still think including malaria and sickle cell to the title makes it a bit misleadinng. I will leave it at the editor's discretion too.*

We are pleased with the positive comments of the reviewers and editor. We address the final remaining comment by revising the title to:

“A high-throughput inhibitor screen for the erythrocyte plasma membrane Ca^++^ ATPase”.
