# Supplementary material for: A high-throughput inhibitor screen for the erythrocyte plasma membrane Ca++ ATPase
Source: PLoS One. 2026 Jun 25;21(6):e0352460. doi: 10.1371/journal.pone.0352460 (PMC13298757; doi:10.1371/journal.pone.0352460)
Supplement: Attachment — Submitted filename: Resp to Reviewers 040726.docx [file pone.0352460.s002.docx]

Response to Reviewers’ comments

We thank both reviewers for their careful and accurate reading of our manuscript and their constructive suggestions. These suggestions have improved presentation of our findings and led to an improved manuscript.

*Reviewer #1: This is a clear-cut, easy to read, interesting and potentially useful original manuscript on the development and optimisation of a novel cell-based high-throughput chemical screen for PMCA enzyme inhibitors using human erythrocytes as model system, based on a Ca2+ efflux assay for the plasma membrane Ca2+ ATPase (PMCA). Moreover, studies have linked PMCA to multiple diseases such as sickle cell disease and malaria severity for the PMCA4b isoform on human erythrocytes and there are no specific known chemical inhibitors of PMCA. The new assay are cheaper than the method with hazardous radioactive Ca2+. Using this method, the authors performed o screening of more than 52,000 small compounds from commercial ChemBridge library and an in-house collection. I especially appreciated that to distinguish true PMCA4b inhibitors from false-positive fluorescence quenchers, the authors added more Ca2+ after the second reading. Ca2+ addition was expected to increase the signal with PMCA4b inhibitors, since it allows to block Ca2+ efflux but the increase would be blunted in wells containing fluorescence quenchers.
Unfortunately, the authors did not find reproducible inhibitors of PMCA4b proteoform. The conclusions are consistent with the evidence and arguments presented.
The manuscript could be particularly useful to researchers interested by this optimized assay in order to identify new PMCA inhibitors as research tools and potential future therapeutics.
The work has been carefully made and edited, and this manuscript doesn’t have any evident suggestion for improvement.
The references are appropriate.
No further comments on the figures.*

We thank this reviewer for their accurate description of our study and their strong positive comments*.*

*Reviewer #2: This study shows the development of an assay that is robust and can be used to screen calcium efflux. Overall, the methodology is sound, and data supports claims of developing a robust method for calcium efflux and compound screening. Despite this, the screen did not yield significant hits for PMCA4b inhibition.
Authors used human red blood cells as their cellular model to study calcium efflux in vitro and screen for inhibitory small compounds. The choice of red blood cells seems reasonable: it’s a relatively accessible cell, simple model and expresses PMCA4b isoform. This isoform is ubiquitously found in other tissues, so this will cover a range of tissues if an inhibitor was found in the screen.
1. PMCA4b has been associated with diseases such as malaria and sickle cell but no work on those diseases were done in this paper. Therefore, Title for manuscript including malaria and sickle cell may present as misleading- this work encompasses mainly developing a miniaturized high scale screening for calcium ion inhibitor. The association of said diseases can be discussed in the text and not be part of the title.*

We consider this an editorial issue and a matter of personal style. We note that many manuscripts in established journals include malaria and/or sickle cell disease in their titles without performing human subjects research, as required for direct study of disease processes. (E.g., PMID: 23958191 from Mohamed *et al* 2013, as mentioned in #2 below, entitled “… calcium ATPase pump … target for contraception and malaria” does not perform either *in vivo* or *in vitro* studies on either contraception or malaria.)

We have not changed our title as it is clear and appropriately describes our study’s motivations. However, if the *PLOS One* editor requires, we can shorten the title to

“A high-throughput inhibitor screen for the erythrocyte plasma membrane Ca^++^ ATPase”.

*2. The study fails to acknowledge aurintricarboxylic acid (ATA), an established PMCA4b inhibitor. ATA has been widely studied as a PMCA inhibitor and it is established as a PMCA4 inhibitor (Mohamed et al 2013). For that reason, lines 7-8, 38-40 does not reflect the literature on PMCA4 inhibitory agent.*

The Mohamed *et al* 2013 paper (PMID: 23880607) used a coupled enzyme ATPase assay to screen 1280 compounds from LOPAC drug-like library and found ATA as an inhibitor with an *IC_50_* of 150 nM against PMCA4. They showed it was less active against PMCA1 and SERCA2a; in their study, ATA was inactive against Na^+^/K^+^ ATPase, Ca^++^ transients in cardiomyocytes, and a GCaMP2 Ca^++^ sensor protein. They concluded it is relatively specific for PMCA4. However, numerous other studies, before and after the Mohamed paper, have found it potently blocks a range of other enzymes, some with higher affinities than for PMCA4: *IC_50_* of < 150 nM for YOP protein tyrosine phosphatase (PMID: 15359123), *IC_50_* of 200 nM for phosphofructokinase (PMID: 2525029), *IC_50_* values of 8.6 nM and 72.9 nM for rP2X1 and rP2X3 receptors (PMID: 31461640). It also is considered a potent inhibitor of ribonucleases in cellular lysates (PMID: 410006) and of multiple steps in protein synthesis (PMID: 5547994).

Resveratrol is another inhibitor that has frequently been used to examine effects of PMCA4 block (PMIDs: 28955914, 35768835, 41400477), but it also has many other targets in cells. Neither compound can be considered sufficiently specific for therapy development. Both compounds appear to be so-called “pan-assay interference compounds” that produce positive results in various screening assays (PMID: 25254460).

To address the reviewer’s concern about lines 7-8, we have revised the Abstract to “there are no PMCA inhibitors with sufficient specificity to serve as starting points for therapy development.” Lines 7-8.

We agree with the reviewer that ATA and resveratrol deserve to be mentioned as chemical inhibitors that have been used in several PMCA studies; both were included in our original paper through citations of key papers that used these chemicals (Souto-Guevara *et al* 2024; Peterson *et al* 2016). To more fully acknowledge these inhibitors and appropriately cite the Mohamed *et al* 2013 and 2016 papers, we have added, “Aurintricarboxylic acid was identified … useful insights in some cases [26, 27].” Lines 40-47.

These compounds do not meet the specificity required for confident examination of cellular or whole-organism phenotypes or the development of safe therapeutics.

*3. Also makes me wonder why ATA was not used as a control in the experiments in addition to the ATPase inhibitor used. This would have shown how, if any hit compound, compares to the established inhibitor (ATA).*

Based on the above specificity observations, we do not see clear advantages of either ATA or resveratrol over vanadate as a blocking control. Vanadate is also nonspecific but it is the generally accepted standard for inhibition of P-type ATPase pumps.

*4. Lines 319-321 also doesn’t necessarily reflect thr literature as functional studies (Zambo et al 2017; Joof et al 2023) has shown effect of mutations on PMCA4 gene on the protein: decreased protein expression and impairment of calcium efflux*

We agree and have revised these statements. See “These mutations … disease processes”, lines 331-339.

The Joof *et al* 2023 paper was already cited in our manuscript; we have added the Zambo study (refs 46 and 47 in the revised paper). We note that both papers relied on erythrocyte loading with Fluo-4-AM and did not examine OAT-mediated dye efflux. Because our Fig. 1A shows rapid efflux of loaded dyes via OATs, it is likely that their measured Ca^++^ fluxes were confounded by dye transport. Both studies also did not attempt to remove added ionomycin, so their measurements include Ca^++^ flux via both PMCA4 and ionophore. The Joof study reported parasite growth inhibition by ATA, but the *IC_50_* was 122 µM, a concentration ~ 1000-fold higher than the compound’s *K_i_* for PMCA4 block, implicating parasite killing through nonspecific effects other targets. These observations suggest that the effects of PMCA4 noncoding mutations on Ca^++^ transport rates are not quantitatively established. Even if these issues are addressed and changes in PMCA4-mediated Ca^++^ transport are better established, it would remain unclear whether *pmca4b* mutations control malaria severity directly through altered Ca^++^ homeostasis or indirectly via effects on cell volume, deformability, or other parameters. With these caveats, we consider it best to interpret these studies cautiously. See “These mutations … disease processes”, lines 331-339.

*5. I like how in the discussion authors suggests other ways of improving the screen of compounds by diversifying libraries and molecular size of compounds. Maybe authors can also comment on the library of chemicals they used from ChemBridge. It is not clear whether 52,000 agents chosen for the screen was systematic in any way. Was it a custom library or just a blind screen?*

Thank you. We designed and selected our libraries for maximal diversity while maintaining drug-like properties with MW ≤ 450 dal and cLogP values ≤ 4.5. Added to the Methods, lines 123-125.

*6. Overall, I think Authors should highlight the strong suits of the study (which is he development of a miniaturized calcium ion assay for high scale screening) and tone down the malaria and sickle cell association of their work. Also, to comment on ATA which is an established PMCA4 inhibitor. Acknowledging the established inhibitor doesn’t take away the need for screening for more inhibitors for PMCA4, which is an important protein in human diseases.*

We agree that the assay and screening technology is the strongest suit of the paper. We have expanded the presentation of known PMCA inhibitors ATA and resveratrol (lines 40-47). We also agree that these inhibitors justify additional screening as they validate our view that PMCA4 is druggable. More specific and potent inhibitors are now within reach through a robust screen that quantitively isolates PMCA4 activity. We have added “PMCA inhibition by aurintricarboxylic acid and resveratrol indicate that effective block by small molecules is indeed possible [16, 21].” Lines 316-317.
