# Supplementary material for: A high-throughput inhibitor screen for the erythrocyte plasma membrane Ca++ ATPase
Source: PLoS One. 2026 Jun 25;21(6):e0352460. doi: 10.1371/journal.pone.0352460 (PMC13298757; doi:10.1371/journal.pone.0352460)
Supplement: Peer review history

# Peer review: Decision Letter 0

**DOI:** 10.1371/journal.pone.0352460.r001

**Authors:** Richard Cardullo

22 Mar 2026

PONE-D-26-05364

A high-throughput inhibitor screen for the erythrocyte plasma membrane Ca++ ATPase linked to malaria and sickle disease

PLOS One

Dear Dr. Desai,

Thank you for submitting your manuscript to PLOS ONE. After careful consideration, we feel that it has merit but does not fully meet PLOS ONE’s publication criteria as it currently stands. Therefore, we invite you to submit a revised version of the manuscript that addresses the points raised during the review process.

Overall, the reviewers were positive regarding this manuscript and feel that it will make a valuable contribution to the literature. Minor modifications are necessary before it can be considered for publication and the comments of both reviewers, especially reviewer 2, should be addressed in a revised manuscript. In particular, reconsider the title of the manuscript given that the paper does not directly address specficic diseases such as malaria or sickle cell anemia. Further, the second reviewer makes a valid point regarding the PMCA4 inhibitor ATA - the manuscript should at least address this compound and why it was not used in this study.

Please submit your revised manuscript by May 06 2026 11:59PM. If you will need more time than this to complete your revisions, please reply to this message or contact the journal office at plosone@plos.org. When you're ready to submit your revision, log on to https://www.editorialmanager.com/pone/ and select the 'Submissions Needing Revision' folder to locate your manuscript file.

Please include the following items when submitting your revised manuscript:

- A letter that responds to each point raised by the academic editor and reviewer(s). You should upload this letter as a separate file labeled 'Response to Reviewers'.
- A marked-up copy of your manuscript that highlights changes made to the original version. You should upload this as a separate file labeled 'Revised Manuscript with Track Changes'.
- An unmarked version of your revised paper without tracked changes. You should upload this as a separate file labeled 'Manuscript'.

If you would like to make changes to your financial disclosure, please include your updated statement in your cover letter. Guidelines for resubmitting your figure files are available below the reviewer comments at the end of this letter.

If applicable, we recommend that you deposit your laboratory protocols in protocols.io to enhance the reproducibility of your results. Protocols.io assigns your protocol its own identifier (DOI) so that it can be cited independently in the future. For instructions see: https://journals.plos.org/plosone/s/submission-guidelines#loc-laboratory-protocols. Additionally, PLOS ONE offers an option for publishing peer-reviewed Lab Protocol articles, which describe protocols hosted on protocols.io. Read more information on sharing protocols at https://plos.org/protocols?utm_medium=editorial-email&utm_source=authorletters&utm_campaign=protocols.

We look forward to receiving your revised manuscript.

Kind regards,

Richard Cardullo, Ph.D.

Academic Editor

PLOS One

**Journal Requirements:**

When submitting your revision, we need you to address these additional requirements.

1. Please ensure that your manuscript meets PLOS ONE's style requirements, including those for file naming. The PLOS ONE style templates can be found at

https://journals.plos.org/plosone/s/file?id=wjVg/PLOSOne_formatting_sample_main_body.pdf and

https://journals.plos.org/plosone/s/file?id=ba62/PLOSOne_formatting_sample_title_authors_affiliations.pdf

2. We note that the grant information you provided in the ‘Funding Information’ and ‘Financial Disclosure’ sections do not match.

When you resubmit, please ensure that you provide the correct grant numbers for the awards you received for your study in the ‘Funding Information’ section.

3. Thank you for stating the following financial disclosure:

“This research was supported by the Division of Intramural Research, NIAID, National Institutes of Health.”

Please state what role the funders took in the study.  If the funders had no role, please state: "The funders had no role in study design, data collection and analysis, decision to publish, or preparation of the manuscript."

If this statement is not correct you must amend it as needed.

Please include this amended Role of Funder statement in your cover letter; we will change the online submission form on your behalf.

4. Please expand the acronym “NIAID” (as indicated in your financial disclosure) so that it states the name of your funders in full.

This information should be included in your cover letter; we will change the online submission form on your behalf.

5. If the reviewer comments include a recommendation to cite specific previously published works, please review and evaluate these publications to determine whether they are relevant and should be cited. There is no requirement to cite these works unless the editor has indicated otherwise.

6. Please review your reference list to ensure that it is complete and correct. If you have cited papers that have been retracted, please include the rationale for doing so in the manuscript text, or remove these references and replace them with relevant current references. Any changes to the reference list should be mentioned in the rebuttal letter that accompanies your revised manuscript. If you need to cite a retracted article, indicate the article’s retracted status in the References list and also include a citation and full reference for the retraction notice.

[Note: HTML markup is below. Please do not edit.]

Reviewers' comments:

Reviewer's Responses to Questions

**Comments to the Author**

1. Is the manuscript technically sound, and do the data support the conclusions?

The manuscript must describe a technically sound piece of scientific research with data that supports the conclusions. Experiments must have been conducted rigorously, with appropriate controls, replication, and sample sizes. The conclusions must be drawn appropriately based on the data presented.

Reviewer #1: Yes

Reviewer #2: Yes

**********

2. Has the statistical analysis been performed appropriately and rigorously?

Reviewer #1: Yes

Reviewer #2: Yes

**********

3. Have the authors made all data underlying the findings in their manuscript fully available?

The PLOS Data policy requires authors to make all data underlying the findings described in their manuscript fully available without restriction, with rare exception (please refer to the Data Availability Statement in the manuscript PDF file). The data should be provided as part of the manuscript or its supporting information, or deposited to a public repository. For example, in addition to summary statistics, the data points behind means, medians and variance measures should be available. If there are restrictions on publicly sharing data—e.g. participant privacy or use of data from a third party—those must be specified.

Reviewer #1: Yes

Reviewer #2: Yes

**********

4. Is the manuscript presented in an intelligible fashion and written in standard English?

PLOS ONE does not copyedit accepted manuscripts, so the language in submitted articles must be clear, correct, and unambiguous. Any typographical or grammatical errors should be corrected at revision, so please note any specific errors here.

Reviewer #1: Yes

Reviewer #2: Yes

**********

5. Review Comments to the Author

Please use the space provided to explain your answers to the questions above. You may also include additional comments for the author, including concerns about dual publication, research ethics, or publication ethics. (Please upload your review as an attachment if it exceeds 20,000 characters)

Reviewer #1: This is a clear-cut, easy to read, interesting and potentially useful original manuscript on the development and optimisation of a novel cell-based high-throughput chemical screen for PMCA enzyme inhibitors using human erythrocytes as model system, based on a Ca2+ efflux assay for the plasma membrane Ca2+ ATPase (PMCA). Moreover, studies have linked PMCA to multiple diseases such as sickle cell disease and malaria severity for the PMCA4b isoform on human erythrocytes and there are no specific known chemical inhibitors of PMCA. The new assay are cheaper than the method with hazardous radioactive Ca2+. Using this method, the authors performed o screening of more than 52,000 small compounds from commercial ChemBridge library and an in-house collection. I especially appreciated that to distinguish true PMCA4b inhibitors from false-positive fluorescence quenchers, the authors added more Ca2+ after the second reading. Ca2+ addition was expected to increase the signal with PMCA4b inhibitors, since it allows to block Ca2+ efflux but the increase would be blunted in wells containing fluorescence quenchers.

Unfortunately, the authors did not find reproducible inhibitors of PMCA4b proteoform. The conclusions are consistent with the evidence and arguments presented.

The manuscript could be particularly useful to researchers interested by this optimized assay in order to identify new PMCA inhibitors as research tools and potential future therapeutics.

The work has been carefully made and edited, and this manuscript doesn’t have any evident suggestion for improvement.

The references are appropriate.

No further comments on the figures.

Reviewer #2: This study shows the development of an assay that is robust and can be used to screen calcium efflux. Overall, the methodology is sound, and data supports claims of developing a robust method for calcium efflux and compound screening. Despite this, the screen did not yield significant hits for PMCA4b inhibition.

Authors used human red blood cells as their cellular model to study calcium efflux in vitro and screen for inhibitory small compounds. The choice of red blood cells seems reasonable: it’s a relatively accessible cell, simple model and expresses PMCA4b isoform. This isoform is ubiquitously found in other tissues, so this will cover a range of tissues if an inhibitor was found in the screen.

1. PMCA4b has been associated with diseases such as malaria and sickle cell but no work on those diseases were done in this paper. Therefore, Title for manuscript including malaria and sickle cell may present as misleading- this work encompasses mainly developing a miniaturized high scale screening for calcium ion inhibitor. The association of said diseases can be discussed in the text and not be part of the title.

2. The study fails to acknowledge aurintricarboxylic acid (ATA), an established PMCA4b inhibitor. ATA has been widely studied as a PMCA inhibitor and it is established as a PMCA4 inhibitor (Mohamed et al 2013). For that reason, lines 7-8, 38-40 does not reflect the literature on PMCA4 inhibitory agent.

3. Also makes me wonder why ATA was not used as a control in the experiments in addition to the ATPase inhibitor used. This would have shown how, if any hit compound, compares to the established inhibitor (ATA).

4. Lines 319-321 also doesn’t necessarily reflect thr literature as functional studies (Zambo et al 2017; Joof et al 2023) has shown effect of mutations on PMCA4 gene on the protein: decreased protein expression and impairment of calcium efflux

5. I like how in the discussion authors suggests other ways of improving the screen of compounds by diversifying libraries and molecular size of compounds. Maybe authors can also comment on the library of chemicals they used from ChemBridge. It is not clear whether 52,000 agents chosen for the screen was systematic in any way. Was it a custom library or just a blind screen?

6. Overall, I think Authors should highlight the strong suits of the study (which is he development of a miniaturized calcium ion assay for high scale screening) and tone down the malaria and sickle cell association of their work. Also, to comment on ATA which is an established PMCA4 inhibitor. Acknowledging the established inhibitor doesn’t take away the need for screening for more inhibitors for PMCA4, which is an important protein in human diseases.

**********

6. PLOS authors have the option to publish the peer review history of their article (what does this mean?). If published, this will include your full peer review and any attached files.

If you choose “no”, your identity will remain anonymous but your review may still be made public.

**Do you want your identity to be public for this peer review?**    For information about this choice, including consent withdrawal, please see our Privacy Policy.

Reviewer #1: No

Reviewer #2: No

**********

[NOTE: If reviewer comments were submitted as an attachment file, they will be attached to this email and accessible via the submission site. Please log into your account, locate the manuscript record, and check for the action link "View Attachments". If this link does not appear, there are no attachment files.]

To ensure your figures meet our technical requirements, please review our figure guidelines: https://journals.plos.org/plosone/s/figures

You may also use PLOS’s free figure tool, NAAS, to help you prepare publication quality figures: https://journals.plos.org/plosone/s/figures#loc-tools-for-figure-preparation.

NAAS will assess whether your figures meet our technical requirements by comparing each figure against our figure specifications.

# Author comment: Author response to Decision Letter 1

**DOI:** 10.1371/journal.pone.0352460.r002

23 Apr 2026

Please see the attached Response to Reviewers document for itemized responses to all queries.

Attachment Submitted filename: Resp to Reviewers 040726.docx

# Peer review: Decision Letter 1

**DOI:** 10.1371/journal.pone.0352460.r003

**Authors:** Richard Cardullo, Richard Cardullo

3 Jun 2026

PONE-D-26-05364R1

A high-throughput inhibitor screen for the erythrocyte plasma membrane Ca++ ATPase linked to malaria and sickle disease

PLOS One

Dear Dr. Desai,

Thank you for submitting your manuscript to PLOS ONE. After careful consideration, we feel that it has merit but does not fully meet PLOS ONE’s publication criteria as it currently stands. Therefore, we invite you to submit a revised version of the manuscript that addresses the points raised during the review process.

Please submit your revised manuscript by Jul 18 2026 11:59PM. If you will need more time than this to complete your revisions, please reply to this message or contact the journal office at plosone@plos.org. When you're ready to submit your revision, log on to https://www.editorialmanager.com/pone/ and select the 'Submissions Needing Revision' folder to locate your manuscript file.

Please include the following items when submitting your revised manuscript:

- A letter that responds to each point raised by the academic editor and reviewer(s). You should upload this letter as a separate file labeled 'Response to Reviewers'.
- A marked-up copy of your manuscript that highlights changes made to the original version. You should upload this as a separate file labeled 'Revised Manuscript with Track Changes'.
- An unmarked version of your revised paper without tracked changes. You should upload this as a separate file labeled 'Manuscript'.

If you would like to make changes to your financial disclosure, please include your updated statement in your cover letter. Guidelines for resubmitting your figure files are available below the reviewer comments at the end of this letter.

If applicable, we recommend that you deposit your laboratory protocols in protocols.io to enhance the reproducibility of your results. Protocols.io assigns your protocol its own identifier (DOI) so that it can be cited independently in the future. For instructions see: https://journals.plos.org/plosone/s/submission-guidelines#loc-laboratory-protocols. Additionally, PLOS ONE offers an option for publishing peer-reviewed Lab Protocol articles, which describe protocols hosted on protocols.io. Read more information on sharing protocols at https://plos.org/protocols?utm_medium=editorial-email&utm_source=authorletters&utm_campaign=protocols.

As the corresponding author, your ORCID iD is verified in the submission system and will appear in the published article. PLOS supports the use of ORCID, and we encourage all coauthors to register for an ORCID iD and use it as well. Please encourage your coauthors to verify their ORCID iD within the submission system before final acceptance, as unverified ORCID iDs will not appear in the published article. *Only*    the individual author can complete the verification step; PLOS staff *cannot*    verify ORCID iDs on behalf of authors.

We look forward to receiving your revised manuscript.

Kind regards,

Richard Cardullo, Ph.D.

Academic Editor

PLOS One

Journal Requirements:

If the reviewer comments include a recommendation to cite specific previously published works, please review and evaluate these publications to determine whether they are relevant and should be cited. There is no requirement to cite these works unless the editor has indicated otherwise.

Please review your reference list to ensure that it is complete and correct. If you have cited papers that have been retracted, please include the rationale for doing so in the manuscript text, or remove these references and replace them with relevant current references. Any changes to the reference list should be mentioned in the rebuttal letter that accompanies your revised manuscript. If you need to cite a retracted article, indicate the article’s retracted status in the References list and also include a citation and full reference for the retraction notice.

**Additional Editor Comments:**

The paper is ready for publication, however, as pointed out by Reviewer 2, the title is misleading in that the study does not involve Sickle Cell or malaria research (although the applications are clear). Please consider a more appropriate title that relates to the actual findings in this study.

[Note: HTML markup is below. Please do not edit.]

Reviewers' comments:

Reviewer's Responses to Questions

**Comments to the Author**

1. If the authors have adequately addressed your comments raised in a previous round of review and you feel that this manuscript is now acceptable for publication, you may indicate that here to bypass the “Comments to the Author” section, enter your conflict of interest statement in the “Confidential to Editor” section, and submit your "Accept" recommendation.

Reviewer #1: All comments have been addressed

Reviewer #2: All comments have been addressed

**********

2. Is the manuscript technically sound, and do the data support the conclusions?

The manuscript must describe a technically sound piece of scientific research with data that supports the conclusions. Experiments must have been conducted rigorously, with appropriate controls, replication, and sample sizes. The conclusions must be drawn appropriately based on the data presented.

Reviewer #1: Yes

Reviewer #2: Yes

**********

3. Has the statistical analysis been performed appropriately and rigorously?

Reviewer #1: Yes

Reviewer #2: Yes

**********

4. Have the authors made all data underlying the findings in their manuscript fully available?

The PLOS Data policy requires authors to make all data underlying the findings described in their manuscript fully available without restriction, with rare exception (please refer to the Data Availability Statement in the manuscript PDF file). The data should be provided as part of the manuscript or its supporting information, or deposited to a public repository. For example, in addition to summary statistics, the data points behind means, medians and variance measures should be available. If there are restrictions on publicly sharing data—e.g. participant privacy or use of data from a third party—those must be specified.

Reviewer #1: Yes

Reviewer #2: Yes

**********

5. Is the manuscript presented in an intelligible fashion and written in standard English?

PLOS ONE does not copyedit accepted manuscripts, so the language in submitted articles must be clear, correct, and unambiguous. Any typographical or grammatical errors should be corrected at revision, so please note any specific errors here.

Reviewer #1: Yes

Reviewer #2: Yes

**********

6. Review Comments to the Author

Please use the space provided to explain your answers to the questions above. You may also include additional comments for the author, including concerns about dual publication, research ethics, or publication ethics. (Please upload your review as an attachment if it exceeds 20,000 characters)

Reviewer #1: This is a clear-cut, easy to read, interesting and potentially useful original manuscript on the development and optimisation of a novel cell-based high-throughput chemical screen for PMCA enzyme inhibitors using human erythrocytes as model system, based on a Ca2+ efflux assay for the plasma membrane Ca2+ ATPase (PMCA). The work has been carefully made and edited, and this manuscript doesn’t have any evident suggestion for improvement.

Reviewer #2: I still think including malaria and sickle cell to the title makes it a bit misleadinng. I will leave it at the editor's discretion too.

**********

7. PLOS authors have the option to publish the peer review history of their article (what does this mean?). If published, this will include your full peer review and any attached files.

If you choose “no”, your identity will remain anonymous but your review may still be made public.

**Do you want your identity to be public for this peer review?**    For information about this choice, including consent withdrawal, please see our Privacy Policy.

Reviewer #1: No

Reviewer #2: No

**********

[NOTE: If reviewer comments were submitted as an attachment file, they will be attached to this email and accessible via the submission site. Please log into your account, locate the manuscript record, and check for the action link "View Attachments". If this link does not appear, there are no attachment files.]

To ensure your figures meet our technical requirements, please review our figure guidelines: https://journals.plos.org/plosone/s/figures

You may also use PLOS’s free figure tool, NAAS, to help you prepare publication quality figures: https://journals.plos.org/plosone/s/figures#loc-tools-for-figure-preparation.

NAAS will assess whether your figures meet our technical requirements by comparing each figure against our figure specifications.

# Author comment: Author response to Decision Letter 2

**DOI:** 10.1371/journal.pone.0352460.r004

9 Jun 2026

We are pleased with the positive comments of the reviewers and editor. We address the final remaining comment by revising the title to:

“A high-throughput inhibitor screen for the erythrocyte plasma membrane Ca++ ATPase”.

Attachment Submitted filename: Response to Reviewers 060926.docx

# Editor report: Decision Letter 2

**DOI:** 10.1371/journal.pone.0352460.r005

**Authors:** Richard Cardullo, Richard Cardullo, Richard Cardullo

10 Jun 2026

A high-throughput inhibitor screen for the erythrocyte plasma membrane Ca++ ATPase

PONE-D-26-05364R2

Dear Dr. Desai,

We’re pleased to inform you that your manuscript has been judged scientifically suitable for publication and will be formally accepted for publication once it meets all outstanding technical requirements.

Within one week, you’ll receive an e-mail detailing the required amendments. When these have been addressed, you’ll receive a formal acceptance letter and your manuscript will be scheduled for publication.

An invoice will be generated when your article is formally accepted. Please note, if your institution has a publishing partnership with PLOS and your article meets the relevant criteria, all or part of your publication costs will be covered. Please make sure your user information is up-to-date by logging into Editorial Manager at Editorial Manager® and clicking the ‘Update My Information' link at the top of the page. For questions related to billing, please contact billing support.

If your institution or institutions have a press office, please notify them about your upcoming paper to help maximize its impact. If they’ll be preparing press materials, please inform our press team as soon as possible -- no later than 48 hours after receiving the formal acceptance. Your manuscript will remain under strict press embargo until 2 pm Eastern Time on the date of publication. For more information, please contact onepress@plos.org.

Kind regards,

Richard Cardullo, Ph.D.

Academic Editor

PLOS One

Additional Editor Comments (optional):

Thank you for your efforts and for changing the title to reflect the contents of the paper.

Reviewers' comments:

# Editor report: Acceptance letter

**DOI:** 10.1371/journal.pone.0352460.r006

**Authors:** Richard Cardullo, Richard Cardullo, Richard Cardullo

PONE-D-26-05364R2

PLOS One

Dear Dr. Desai,

I'm pleased to inform you that your manuscript has been deemed suitable for publication in PLOS One. Congratulations! Your manuscript is now being handed over to our production team.

At this stage, our production department will prepare your paper for publication. This includes ensuring the following:

* All references, tables, and figures are properly cited

* All relevant supporting information is included in the manuscript submission,

* There are no issues that prevent the paper from being properly typeset

You will receive further instructions from the production team, including instructions on how to review your proof when it is ready. Please keep in mind that we are working through a large volume of accepted articles, so please give us a few days to review your paper and let you know the next and final steps.

Lastly, if your institution or institutions have a press office, please let them know about your upcoming paper now to help maximize its impact. If they'll be preparing press materials, please inform our press team within the next 48 hours. Your manuscript will remain under strict press embargo until 2 pm Eastern Time on the date of publication. For more information, please contact onepress@plos.org.

You will receive an invoice from PLOS for your publication fee after your manuscript has reached the completed accept phase. If you receive an email requesting payment before acceptance or for any other service, this may be a phishing scheme. Learn how to identify phishing emails and protect your accounts at https://explore.plos.org/phishing.

If we can help with anything else, please email us at customercare@plos.org.

Thank you for submitting your work to PLOS ONE and supporting open access.

Kind regards,

PLOS ONE Editorial Office Staff

on behalf of

Dr. Richard Cardullo

Academic Editor

PLOS One
